# Advances in the Research of Melatonin in Autism Spectrum Disorders: Literature Review and New Perspectives

**DOI:** 10.3390/ijms141020508

**Published:** 2013-10-14

**Authors:** Sylvie Tordjman, Imen Najjar, Eric Bellissant, George M. Anderson, Marianne Barburoth, David Cohen, Nemat Jaafari, Olivier Schischmanoff, Rémi Fagard, Enas Lagdas, Solenn Kermarrec, Sophie Ribardiere, Michel Botbol, Claire Fougerou, Guillaume Bronsard, Julie Vernay-Leconte

**Affiliations:** 1 Hospital-University Department of Child and Adolescent Psychiatry, Guillaume Régnier Hospital, Rennes 1 University, Rennes 35000, France; E-Mails: imen.najjar@gmail.com (I.N.); inas-lagdas@hotmail.com (E.L.); solennkermarrec@yahoo.fr (S.K.); sophie.ribardiere@yahoo.fr (S.R.); julie9175@hotmail.fr (J.V.-L.); 2 Laboratory of Psychology of Perception, CNRS UMR 8158, Paris 75270, France; E-Mail: marianne.barburoth@parisdescartes.fr; 3 Inserm CIC 0203 Clinical Investigation Centre, University Hospital, Rennes 1 University, Rennes 35033, France; E-Mails: eric.bellissant@chu-rennes.fr (E.B.); claire.fougerou@chu-rennes.fr (C.F.); 4 Department of Clinical Pharmacology, University Hospital, Rennes 1 University, Rennes 35033, France; 5 Laboratory of Developmental Neurochemistry, Yale Child Study Center, New Haven, CT 06519, USA; E-Mail: george.anderson@yale.edu; 6 Department of Laboratory Medicine, Yale University School of Medicine, New Haven, CT 06519, USA; 7 Hospital-University Department of Child and Adolescent Psychiatry, Pitié-SalpétrièreHospital, Paris 6 University, Paris 75013, France; E-Mail: david.cohen@psl.aphp.fr; 8 CIC INSERM U 802, CHU de Poitiers, Unité de recherche clinique intersectorielle en psychiatrie du Centre Hospitalier Henri Laborit, Poitiers 86022, France; E-Mail: nemat.jaafari@ch-poitiers.fr; 9 INSERM UMR U978, University of Paris 13, Bobigny 93009, France; E-Mails: olivier.schischmanoff@avc.aphp.fr (O.S.); remi.fagard@avc.aphp.fr (R.F.); 10 Laboratoire de Biochimie et Biologie Moléculaire, Hôpital Avicenne, APHP, Bobigny 93009, France; 11 Service Hospitalo-Universitaire de Psychiatrie de l’Enfant et de l’Adolescent de Brest, UBO, Brest 29238, France; E-Mail: michel.botbol@orange.fr; 12 Maison Départementale de l’Adolescent et Centre Médico-Psycho-Pédagogique, Conseil Général des Bouches-du-Rhône; Laboratoire de Santé Publique EA3279, Faculté de Médecine de la Timone, Marseille 13256, France; E-Mail: guillaume.bronsard@free.fr

**Keywords:** melatonin, biological clocks, circadian rhythm, synchrony, autism spectrum disorders, social communication, stereotyped behaviors

## Abstract

Abnormalities in melatonin physiology may be involved or closely linked to the pathophysiology and behavioral expression of autistic disorder, given its role in neurodevelopment and reports of sleep-wake rhythm disturbances, decreased nocturnal melatonin production, and beneficial therapeutic effects of melatonin in individuals with autism. In addition, melatonin, as a pineal gland hormone produced from serotonin, is of special interest in autistic disorder given reported alterations in central and peripheral serotonin neurobiology. More specifically, the role of melatonin in the ontogenetic establishment of circadian rhythms and the synchronization of peripheral oscillators opens interesting perspectives to ascertain better the mechanisms underlying the significant relationship found between lower nocturnal melatonin excretion and increased severity of autistic social communication impairments, especially for verbal communication and social imitative play. In this article, first we review the studies on melatonin levels and the treatment studies of melatonin in autistic disorder. Then, we discuss the relationships between melatonin and autistic behavioral impairments with regard to social communication (verbal and non-verbal communication, social interaction), and repetitive behaviors or interests with difficulties adapting to change. In conclusion, we emphasize that randomized clinical trials in autism spectrum disorders are warranted to establish potential therapeutic efficacy of melatonin for social communication impairments and stereotyped behaviors or interests.

## 1. Introduction

Melatonin is a neurohormone well known for its effect on the regulation of the circadian sleep-wake rhythm. In physiologic conditions, its plasmatic concentration follows a circadian rhythm, with low levels during the day and high levels at night; in humans, the peak secretion is typically occurring at around 2 AM and melatonin has been called the darkness hormone [1].

Since its discovery [2], the biosynthesis and molecular action of melatonin (5-methoxy-*N*-acetyltryptamine) have been thoroughly studied. Melatonin is mainly synthesized by the pinealocytes in the pineal gland [3]. This synthesis is entrained by ambient light under the control of the circadian clock located in the suprachiasmaic nuclei (SCN) of the hypothalamus [4]. Melatonin is produced from the amino acid tryptophan which hydroxylated and then decarboxylated to form 5-hydroxytryptamine or serotonin. Serotonin is first acetylated through the action of the (typically) rate-limiting arylalkylamine-*N*-acetyltransferase (AA-NAT, also termed “Timezyme”), and then *O*-methylated by acetylserotonin *O*-methyltransferase (ASMT) to yield melatonin [5]. Both AA-NAT and ASMT activities are controlled by noradrenergic and neuropeptidergic projections to the pineal gland [6]. Once synthesized, melatonin is immediately released into the systemic circulation to reach peripheral and central target tissues. At this level, the melatonin distributes a nocturnal/circadian message within the entire body to regulate daily and seasonal physiological rhythms through three different molecular pathways. The most well characterized pathway is the binding and activation of the membrane specific G protein-coupled melatonin receptors MT1 andMT2 as well binding to the MT3 site shown to be the enzyme quinone reductase 2) [7]. It appears that melatonin also interacts with nuclear receptor and intracellular proteins [8]. In the liver, melatonin is rapidly metabolized to 6-hydroxymelatonin by the action of the cytochrome P450 enzyme CYP1A2, leading to a short half-life in the circulation (20 to 40 min). Plasma levels can be directly measured or indirectly assessed through salivary measures. Production of melatonin over time can be assessed via measurement of its inactive urinary metabolite, 6-sulphatoxymelatonin (sulfated 6-hydroxymelatonin).

The crucial role of melatonin as a modulator of sleep is well documented. First, disturbances in melatonin levels have been documented with regard to circadian rhythm sleep disorders [1]. Second, administration of exogenous melatonin has been reported to improve sleep-wake rhythm disturbances and to affect sleep latency [9–11]. In a recent experimental study, authors demonstrated that, depending on the activated melatonin receptors (MT1, MT2 or both) melatonin regulates differently the vigilance states: MT2 receptors are mainly involved in non-rapid eye movement sleep, whereas MT(1) receptors are mainly involved in rapid eye movement sleep [12]. In addition to its influence on the daily sleep-wake cycle, melatonin has a large spectrum of effects [13]. At an experimental level, melatonin has been shown to influence basal metabolism, oxidative stress, inflammation, apoptosis [14–18] and to prevent premature aging and tumorigenesis [19]. Interestingly, in humans and animals, melatonin seems to have also an influence on cognitive functions. In mice models of Down syndrome (Ts65Dn mice), melatonin reduces neurodegenerative processes and improves cognitive abilities [20]. In rats with epilepsy, melatonin treatment improved deficits in hippocampus-dependent working memory and behavioral alterations associated with hyperactivity [21]. Finally, melatonin has been reported to significantly improve cognitive abilities and mood in patients with mild cognitive impairment [22].

The effect of exogenously administered melatonin on the circadian clock (chronobiotic properties) and circadian activity rhythms has been documented [23–25]. More recently, its role as a neuroendocrine synchronizer of molecular oscillatory systems has been studied [26,27]. The results provide evidence that melatonin plays a major role in the circadian oscillatory rhythms observed in the expression of several clock genes, such as *PER1*, *PER2*, *BMAL1*, *REV-ERBα*, *CLOCK* and *CRY1*, in both central and peripheral melatonin target tissues. Indeed, in animal models, *PER1* expression is undetectable in melatonin-deficient mice [28,29] and pinealectomy abolishes rhythmic expression of *PER1* in the *Pars tuberalis* (PT) [30] and results in desynchronized *PER1* and *PER2* expressions in the SCN [31]. Moreover, it was recently reported that the mutation of *PER1* in mice significantly altered the rhythms of cytokine and cytolytic factors in splenic natural killer (NK) cells resulting in altered rhythms of NK cellular clocks and immune pathways [32]. Melatonin synchronizes circadian oscillations in the cardiovascular system by influencing circadian rhythmic expression of both *PER1* and *BMAL1* in the rat heart [33]. In adipose tissue, melatonin synchronizes metabolic and hormonal function [34] by regulating *PER2*, *CLOCK* and the nuclear receptor *REV-ERBα* [35]. The latter is essential for the daily balance of carbohydrate and lipid metabolism [36]. Finally, melatonin regulates oscillation of *CLOCK* genes in healthy and cancerous human breast epithelial cells [37], induces a shift in the 24-h oscillatory expression of *PER2* and *BMAL1* in cultured fetal adrenal gland [38] and influences rhythmic circadian modulation protein synthesis in hepatocytes [39] and erythrocytes [40].

Taken together, these studies underline the major role of melatonin in the regulation of human circadian rhythms including the sleep-wake, neuroendocrine and body temperature cycles [41,42], and more specifically in the synchronization of peripheral oscillators (*i.e.*, in the adjustment of the timing of existing oscillations). Measures of melatonin are considered the best peripheral indices of human circadian timing [43].

Finally, there is increasing evidence that melatonin is critically involved in early development through its direct effects on placenta, developing neurons and glia, and its role in the ontogenetic establishment of diurnal rhythms [44,45]. This strengthens interest in the study of melatonin in developmental disorders, in particular when these developmental disorders are associated with alterations in the sleep-wake cycle. Autistic disorder—a pervasive developmental disorder characterized by communication and social interaction impairments associated with repetitive interests and behaviors—provides an interesting and challenging model of abnormal melatonin production in early developmental disorders and its possible relationships with autistic behavioral impairments. This model offers promising avenues, developed in the next sections, for potential therapeutic benefits of melatonin and a better understanding of its role in social communication development, as a synchronizer and regulator of the circadian rhythms network.

## 2. Melatonin in Autism

Melatonin is of interest in autism spectrum disorder (ASD) due to its apparent role in neurodevelopment [46] and reports of sleep-wake rhythm disturbances in individuals with autism [47]. More specifically, reduced total sleep and longer sleep latency as well as nocturnal and early morning awakenings, are often reported for individuals with ASD [48–55]. In addition, central and peripheral alterations in serotonin in autism have been widely reported and, as mentioned, melatonin is synthesized in only two steps from serotonin in the pineal gland and the gut. [53,56,57].

Prior studies of melatonin production in autistic disorder were often limited by small sample sizes and were not entirely consistent, but all reported abnormalities in the melatonin production (see Table 1). Our results [58,59], taken together with the other studies, strongly indicate that nocturnal secretion of melatonin is often low in autism. It is noteworthy that melatonin abnormalities have been found in several other disorders with intellectual disability [60,61], raising the issue of the non-specificity of the melatonin findings in ASD. However, melatonin production in Down syndrome has been reported to be normal, while increased levels have been reported for Fragile X individuals [62,63].

Furthermore, nocturnal excretion of 6-SM was significantly negatively correlated with severity of autistic impairments in verbal communication and play [58,59]. In addition, our study [58] conducted on 43 individuals with autism, showed low daytime excretion of melatonin in autism that is consistent with Melke *et al.*’s study [67], but contrasts with previous smaller studies of urinary melatonin [64] and serum melatonin [65] reporting higher daytime levels in autism. Our findings [59] taken together with Melke *et al.*’s findings [67] demonstrate that there is a deficit in melatonin production in a substantial proportion of individuals with autism and this deficit is present at night and during the day, indicating that pineal and, possibly, extra-pineal production of melatonin is lower in autism. A potential contribution of the ASMT enzyme to the observed reduced melatonin production in ASD has been considered given that several of the identified mutations of the ASMT gene reduce or abolish ASMT activity (the allelic frequency of ASMT deleterious mutations ranges from 0.66% in Europe to 2.97% in Asia) [69]. Melke *et al*. [67] identified mutations and Cai *et al*. [70] identified microduplications in ASMT, possibly leading to a decrease in ASMT activity in individuals with ASD. However, the ASMT findings of Melke *et al.* have not been replicated and ASMT mutations have been also found in healthy individuals [71,72]. In addition, mutations altering the functional properties of melatonin receptors have been found in individuals with ASD. Although mutations in MT1 and MT2 receptors do not represent major risk factors for ASD [73], they should also be examined as potential contributors to altered melatonin physiology in ASD. Finally, environmental factors occurring at a critical period of development and interacting with genetic factors might contribute to low melatonin levels in ASD. Thus, summer birth was found to be a risk factor for autism according to the 2011 Gardener *et al*. [74] meta-analysis conducted on 64 studies (summer birth was significantly associated with an elevated risk of autism, RR = 1.14, *p =* 0.02). Given that the summer season corresponds to the longest days of the year, a possible role of an early deficit in melatonin in the development of ASD could be speculated (the production of melatonin is powerfully suppressed by light acting through the retino-hypothalamic tract [75]).

Our result of smaller intra-individual 6-SM nighttime-daytime differences and the absence of melatonin variation found in some individuals with autism [59] might be a reflection of the lower day and nighttime levels, but might also indicate that there exists a subgroup of individuals with autism that have a dysregulation of their circadian rhythm, and more precisely an absent circadian rhythm. The hypothesis of an absent circadian rhythm in melatonin and other neuroendocrine functions is supported by Kulman *et al.*’s study [66] in which 10 out of 14 children with autistic disorder showed no melatonin circadian variation (see Table 1), by Zapella [76] who found a blunted circadian rhythm of melatonin secretion in a male adolescent with autism and hypomelanosis of Ito, and by several reports in autism of abnormalities in the circadian rhythm of cortisol secretion including an attenuated circadian variation (see the Tordjman *et al*. review [77]). The possible existence and characteristics of a subgroup of patients with autism showing a deficit in melatonin production with no nighttime-daytime variations may be fruitfully examined in future larger studies.

Finally, beneficial effects of melatonin when administered to individuals with autism and sleep problems have been reported and strengthen the interest to study melatonin in autism [78–80]. These studies are reviewed in the next section.

## 3. Treatment Studies of Melatonin in Autistic Spectrum Disorders

A number of case studies and therapeutic trials of melatonin focused on individuals with developmental disorders and sleep problems have been reported. The main results of these studies, as well as relevant reviews and meta-analyses, are presented in Tables 2,3.

Although potential therapeutic use of melatonin for sleep problems in autism have been considered for many years [108], available data in large samples of children with autistic disorder are very limited (see Table 2). Indeed, the studies have been hampered by small sample sizes of children with autism [81–84,86,87,89,94,96], or have been conducted on larger sample sizes of children with heterogeneous developmental disorders (children with autism mixed with blind children and/or children with multiple neurological disabilities associated with intellectual disability) with no indication with regard to the specificity of the results for the autism group [88,91,95–99]. As already indicated, there is an issue of potential non-specificity of the melatonin findings in ASD, especially given that melatonin is widely used as a treatment in other disorders associated with intellectual disability [96,109]. Further studies are required to test better the melatonin findings with regard to ASD or intellectual disability. An alternative hypothesis would be that the melatonin findings are related to impairments in certain dimensions, such as the communication or social domain, shared by several different disorders. Finally, it cannot be ruled out that the melatonin findings are related to sleep-wake rhythm disturbances without any specificity with regard to a particular disorder. However, it is noteworthy that in our studies [58,59], melatonin deficit was significantly associated with social communication impairments, but not with sleep problems. Future research on melatonin in ASD is required, including therapeutic trials, studying together melatonin levels, sleep problems, autistic behavioral impairments and cognitive level of functioning in order to better understand the relationships between these variables.

Another major issue for clinical trials of melatonin in ASD is the loss of response to melatonin treatment underlined by Braam and colleagues for patients with intellectual disability and sleep problems, including patients with ASD [110,111]. In addition, Andersen *et al*. [85], in a retrospective study on 107 children with ASD that were prescribed melatonin for insomnia, found seven cases in which sleep initially improved, but sleep problems returned, despite dose escalation. Braam *et al*. [110,111] suggest that loss of response in these patients might be a result of exposure to persistent non-physiologic melatonin levels due to slow melatonin metabolism provoked by decreased activity of CYP1A2. In the case of loss of response, Braam *et al*. [110,111] recommend that melatonin dose should be greatly reduced, rather than increased.

Furthermore, only a few of the therapeutic trials report results related to outcomes other than sleep, in particular autistic behavioral impairments. Thus, improvement of communication [97], social withdrawal [90,92], stereotyped behaviors and rigidity [92,94] or anxiety [90,95] was reported in children with ASD using melatonin. The relevant studies are detailed in Table 2. In addition, Jan and Freeman [107] discussed, based on their literature review of studies using dose escalation from 1–2 mg up to 15 mg, that the higher melatonin doses were reached in children with greater mental or motor comorbidities. It is noteworthy that several dose escalation studies were conducted (see Table 2), but there is currently no study of the dose-response relationship for melatonin in ASD. The strengths of the treatment studies mentioned above [90,92,94,95,97] are their interest for autistic behavioral impairments and their design which includes controlled trials of melatonin for all of them and randomized double-blind, placebo-controlled trials for most of them [94,95,97]. However, some limitations can be noted. Thus, ASD populations studied [90,95,97] were often clinically heterogeneous (for example, autistic disorder according to DSM-IV-TR criteria can be mixed with pervasive developmental disorder not otherwise specified). The age range was very wide in some studies and prepubertal children were mixed with pubertal or postpubertal individuals [94,95,97]. This is a problem given that pineal melatonin secretion is influenced by age and pubertal stage [112,113]. In addition, autistic behavioral impairments were often not detailed enough (for example, Wright *et al.* [97] reported significant communication improvement but the results were not detailed with regard to verbal or non-verbal communication) or were not always assessed using validated tools (for example, Garstang and Wallis [94] reported decreased rigidity based only on parents’ and teachers’ comments and Wasdell *et al.* [95] reported decreased anxiety based on caregivers’ comments). Finally, it is noteworthy that no clinical trial of melatonin in autistic disorder used behavioral autistic impairments as their main outcome criteria. Further therapeutic trials of melatonin are necessary, conducted on large samples of prepubertal children with autistic disorder and using validated behavioral assessments, to study better the evolution of autistic behavioral impairments following administration of melatonin.

## 4. Relationships between Melatonin and Autistic Behavioral Impairments

The relationships between melatonin and autistic behavioral impairments are discussed below with regard to the three main DSM-IV-TR domains of autism (communication including verbal/non-verbal communication, reciprocal social interactions, and restricted, repetitive and stereotyped behaviors or interests with difficulties adapting to change) and the role of synchrony of rhythms in social communication development.

### 4.1. Melatonin and Communication

#### 4.1.1. Melatonin and Autistic Communication Impairments

As seen in the previous section, some studies suggest that administration of melatonin to individuals with autistic disorder improves their communication deficit [97]. Furthermore, we found that nocturnal excretion of 6-sulphatoxymelatonin (6-SM) was significantly negatively correlated with severity in the overall level of verbal language [59]. This observation of significant negative correlations between nocturnal 6-SM excretion and severity of autistic impairment in verbal communication replicates our previous finding [58]. It is noteworthy that we replicated this result using the ADI-R (the ADI-R is based on a parental interview) which differs from the ADOS (the Autism Diagnostic Observation Schedule is based on a direct observation of the patient [114]) used in our previous study. The significant positive correlation between verbal IQ scores and nocturnal 6-SM excretion found in the Tordjman *et al*. study [58] goes in the same direction, although the range of IQ scores in the patients was too narrow to thoroughly test the relationship between IQ scores and 6-SM levels. The observed correlations between severity of communication impairments and decreased nocturnal 6-SM excretion might be related to previous reports of an association between communication impairments and problems with sleep onset in low-functioning children [115]. Thus, some authors suggest that problems of sleep initiation and maintenance in children with ASD may alter their cognitive development, including memory, learning and communication [116]. Other authors suggest that language performance displays an internally generated circadian rhythmicity following the sleep-wake rhythm (the optimal time for parsing language would occur between 3 to 6 h after the habitual wake time) [117]. However, the results obtained in our first study [58] did not indicate that melatonin excretion was closely associated with degree of sleep disturbance in children with autism and using a brief sleep assessment we did not observe sleep problems in the post-pubertal subjects of our second study [59]. It is noteworthy that melatonin has been reported to affect the temporal organization of the song of the Zebra Finch [118]. However, it should also be noted that the circadian pattern of song production was not altered, suggesting that melatonin might be able to influence social behavior through non-circadian pathways. In addition, Nir and colleagues [65] presented data suggestive of reduced melatonin production in individuals with autism and speech difficulties or with EEG abnormalities. More compelling is the agreement between our finding of a negative relationship of nocturnal 6-SM excretion with severity of language impairment and the study by Hu and colleagues [119] in ASD that reported substantially reduced expression of the gene encoding arylalkylamine *N*-acetyltransferase (AA-NAT, the rate limiting enzyme for melatonin synthesis) in ASD individuals with severe language impairment. Finally, the lower mean melatonin production, the significantly smaller day-night differences and the significantly higher frequency of absence of circadian variation observed in individuals with autism compared to controls, might be playing a role in, or be a reflection of, the hypothesized timing problems in “biological clocks” in autism [120,121]. Indeed, melatonin signals can drive daily rhythmicity and are also involved in the synchronization of peripheral oscillators [26]. Thus, Boucher suggests that timing problems in “biological clocks” would have physiological and psychological consequences that might be involved in autistic impairments. Wimpory *et al*. [121] have theorized that anomalies in clock genes operating as timing genes in high frequency oscillator systems may underline timing deficits that could be important in the development of autistic disorder, notably in autistic communication impairment.

#### 4.1.2. Role of Synchrony of Rhythms in Communication Development

The role of melatonin on daily rhythmicity and synchronization of rhythms suggests that melatonin might be involved in motor and emotional synchrony. In line with the hypothesis of ergodicity [122] that postulates the existence of similar mechanisms at different levels, relationships might exist between cellular communication networks involving a cellular synchrony (synchronization of cellular oscillations) and early communication development involving a synchrony of motor and emotional rhythms. This provides a new perspective for considering the relationships between melatonin and communication. Abnormal melatonin production might impair the development of communication as well as socialization (see next section), two main domains of autistic disorder. It is noteworthy that congenitally blind children with consequently abnormal melatonin secretion and synchronization, very frequently meet criteria for autistic disorder (up to 42% [123]), whereas hearing impaired children, including hearing loss, meet criteria less frequently (up to 10% [124]).

### 4.2. Melatonin and Social Interaction

#### 4.2.1. Melatonin and Autistic Social Interaction Impairments

Administration of melatonin was reported in two studies [90,92] to improve social withdrawal in children with ASD. In addition, nocturnal excretion of 6-SM was significantly correlated with severity of autistic social interaction impairments, in particular with imitative social play assessed in a study using the ADI-R [59] and with play assessed in another study using the ADOS [58]. These results, taken together with the importance of synchrony of rhythms in the development of social imitation and very early social interaction, suggest a possible role of melatonin in autistic social interaction impairments.

#### 4.2.2. Role of Synchrony of Rhythms in Social Interaction Development

Concerning the development of social interaction, it is important to highlight the major role of synchrony of rhythms in bonding. Thus, Guedeney *et al*. [125] emphasize the importance of synchronization between infant and parental rhythms in very early social interaction and socio-emotional development, from biological rhythms during pregnancy to later exchange between caregiver and child.

Human learning and cultural evolution are supported by paradoxical biological adaptation. We are born immature; yet, immaturity has value: “*Delaying maturation of cerebral cortex allows initial learning to influence the neural architecture in ways that support later*, *more complex learning*” [126]. Early learning appears to be computational [127] and to be based on perceptual-action mapping [126]. Learning is also social [128] and supported by skills present in infancy: imitation, shared attention and empathic understanding [126]. The whole social system which contributes to interactional synchrony may be disrupted in infant who will subsequently develop autism. It is likely that an atypical social trajectory in the infant would affect parents’ interactive patterns. Temporally, the interactive nature of human social relationships implies that a message a_i_ produced by A impacts B who, in return, produces message b_i_ and so on, indicating that some form of reciprocity occurs between partners A and B [129]. Synchrony is difficult to define and delimit. Numerous terms have been used to describe the interdependence of dyadic partners’ behaviors (mimicry, social resonance, coordination, synchrony, chameleon effect, *etc.*). Here, we define synchrony as the dynamic and reciprocal adaptation of the temporal structure of behaviors between interactive partners [130]. In typically developing children, the quality of social interaction depends on an active dialogue between the parent and the infant based on the infant’s desire to be social and the parent’s capacity to be attuned [131,132]. Numerous studies have emphasized the importance of synchrony and co-modality [133]. Also, synchrony between partners has been correlated with biological markers. Dumas *et al*. [134] use hyper-scanning recordings to examine brain activity, including measures of neural synchronization between distant brain regions of interacting individuals through a free exchange of roles between the imitator and the model. Their study was the first to record dual EEG activity in dyads of subjects during spontaneous nonverbal interaction. Five female-female pairs and 6 male-male pairs were scanned. They showed that interpersonal rhythmic oscillations were correlated with the emergence of synchronization in the brain’s alpha-mu band (an area involved in social interaction [135]) between the right centro-parietal regions. Correlation at biological levels has also been found. Naturally occurring variations in maternal behavior are associated with differences in estrogen-inducible central oxytocin receptors, which are involved in pro-social behaviors [136]. Oxytocin appears to enhance both maternal/paternal as well as affiliative behaviors in humans and is considered as the bonding hormone [137].

These developments have prompted developmental psychologists to study early interaction not only as the addition of two behaviors but rather as a single phenomenon with a dialogue between two partners engaged in behavioral and emotional exchange. Rhythm, synchrony and emotion are increasingly being viewed by developmental psychologists as key aspects of appropriate early interaction [133].

Very few studies have addressed the importance of infant-caregiver synchrony/reciprocity in early interactions involving infants who will subsequently develop autism. Studies using early home videos [138,139] parental interviews [140] and prospective assessment of siblings of children with ASD [141,142] have revealed atypical developmental tendencies in infants who were later diagnosed with ASD. The first signs are abnormalities with eye contact, imitation, disengagement, joint attention, orienting to name, and body language. These behaviors constitute important precursors of later-developing symptoms. However, whether these first signs impact the interactive process between an infant and their parents and whether they influence the development of the infant himself remain two complex and unexplored issues. In two related studies based on home movies, Saint-Georges *et al.* [138] and Cohen *et al.* [143] showed that when studying interactive patterns in infants with computational methods to take into account motor and emotional synchrony between partners: (i) deviant autistic behaviors appeared before 12 months; (ii) parents felt weaker interactive responsiveness and mainly weaker initiative from their infants; (iii) parents tried increasingly to supply soliciting behaviors through touching; fathers’ involvement in interacting with infants that will develop autism significantly increased after 12 months compared to typical developing infants. It is likely that these modifications of interactive patterns implicate numerous co-influences due to the reciprocal nature of these processes.

In fact, it is difficult to separate social from communication development given that emotional synchrony, as well as imitation, play a role in both domains. It might be more appropriate, at this point, to consider the combined domain of social communication as does the most recent version of the scale ADOS [114], as well as the recently released DSM-V. Thus, many studies have referred to the difficulties observed in children with autism in imitating other people’s faces, gestures or vocal signals to better understand their problems with social interaction and speech development. This specific type of imitation is referred to as “spatial” imitation to highlight the capacity to produce an instantaneous copy of the form of the signal. However, another way to communicate and interact with others is to perform a “temporal” imitation of their behavior [144]. This is what humans do when singing, dancing, foot tapping or drumming in synchrony with others. The uniqueness of temporal imitation is that there is no need to use the same motor pattern to succeed in communicating and interacting: the synchronization can occur in movements as simple as finger tapping in synchrony with another person’s body swaying, foot tapping in response to complex songs, or even eye blinking. While both animals and humans are able to perceive rhythms and produce rhythmic motor patterns, the capacity to adapt the rhythm of their movements to an external auditory or visual rhythm is unique to humans [145] (except in the cockatoo, a non-human vocal learning species [146]). Being rhythmically synchronized with his/her environment is crucial for an infant’s emotional, cognitive, social and sensorimotor development [147,148]. Developmental studies have shown that the ability to perceive, as seen previously, and produce rhythms is already present in the human fetus and newborn [149], Barburoth *et al.*, in preparation. However, the capacity to produce temporally-adapted motor patterns emerges later, depending on the motor system used (sucking, finger or hand movement) and the difference between the beat and the spontaneous motor tempo of the infant’s movement [150,151]. Further research is requested to explore the development of children with autism with regard to their capacity to adapt their own rhythm to an external rhythm. Previous studies suggest disorganized rhythms are associated with stereotypies and poor synchrony in these children [152]. Given this background, it is remarkable that melatonin levels have been reported to be low in individuals with autism and negatively correlated with the severity of verbal communication and social imitative play impairments. It could be hypothesized that melatonin, as a regulator of biological rhythms, could enhance the capacity of children with ASD to produce motor-temporal imitation. In this case, administration of melatonin could help them to synchronize their movements with movements of others (playing situations) and with external rhythmic auditory stimuli, such as music and/or the human voice (enhancing their verbal skills). Furthermore, administration of melatonin may change the spontaneous motor tempo of treated children’s movements and enlarge the scope of adaptability of these movements to external stimuli. A third hypothesis is that melatonin treatment may increase the perception of rhythms in children with autism. All of these hypotheses need to be tested and open new avenues for exciting research in the field of synchronization and social communication in ASD.

### 4.3. Melatonin and Restricted, Repetitive and Stereotyped Behaviors or Interests

#### 4.3.1. Melatonin and Stereotyped Behaviors

Repetitive behaviors can be speculated to be an attempt to produce repeated sequences in order to compensate for a lack of daily rhythmicity and synchronized rhythms due to the low melatonin production in ASD. Our finding [59] observed in a sample of 43 adolescents and young adults with autistic disorder (nocturnal excretion of 6-SM was significantly negatively correlated with repetitive use of objects) taken together with the improvement of stereotyped behaviors reported following administration of melatonin in 24 children and adolescents with ASD [92] lend some support to this hypothesis.

Donald Winnicott [153] emphasized that “*the main problem for a typically developing child is to be able to create a continuum out of discontinuity*”. According to him, the first optimal container (“holding environment”) for the newborn is the progressive internalization of the rhythmic structures of feeding (rhythmic ebb and flow corresponding to the kinesthetic experience of suckling), providing a sense of continuing existence. We can nevertheless hypothesize that this internalization process starts far earlier, in the womb. It is through the regular repetition of identical sequences of discontinuity, such as the circadian rhythms that are already present during the fetus life, that a continuum is constructed, together with the sense of continuing existence. Conversely, based on clinical observations in autism, we could say that children with autism create discontinuity out of continuity. Many of these children need to create discontinuity that is repeated at regular intervals, which could have been fundamentally lacking in their physiological development. This overriding need can be observed when children with autism are exposed to stress. It may also occur when they are confronted with a representation of continuity (drawing of a circle, prolonged wait with inactivity, *etc.*). It is at these times that we often witness the emergence of behavioral stereotypies, consisting of body oscillations, repetitive utterances or, for those with graphic skills, the compulsion to write out sequences of numbers. These compulsions might arise from obsessive mechanisms involving a need for control and perhaps, above all, an existential need to produce discontinuous but equidistant patterns. Sylvie Tordjman [154] reported the case of a high-functioning adult with autism, a patient of René Diatkine (filmed sessions providing an extremely interesting and scientifically significant account [155]), who complained of being assailed by a constant flow of thoughts and was only able to overcome his anxiety by ending all his sentences with a pause. When he was a child, this same patient could not stand circles and spent his time producing geometric figures made up of broken lines. The irregular, if not arrhythmic, circadian rhythms reported in some individuals with autism suggest that the development of children with autism is based not on regularly repeated physiological discontinuities, but on anarchic discontinuities and even, in many cases, on an “endless” physiological continuity provoked by the absence of variation in melatonin levels.

Oscillatory, pendular or vortical rhythmic structures appear to be the form, expression and representation of the life instinct and life drive in their most biological essence [156]. Identical patterns, through a stable rhythm repeated at regular intervals, allow us to face up to anxiety about definitive loss and disappearance, and therefore to face up to fears of death. Conversely, it is when this internal rhythm is absent that we witness the emergence of motor stereotypies and ruminations in individuals with autism, as indicated by our results [59]. We can state the hypothesis that children with autism, confronted with this “endless” physiological continuity (or, at the very least, with this absence of physiological discontinuities repeated at regular intervals), develop disorganizing existential anxieties and therefore they try to control them through stereotyped behaviors. Thus, they may become completely absorbed in unimodal stereotyped behaviors involving the overloading of a sensory channel (auditory, visual, kinesthetic, tactile, *etc.*) that enables them to be totally centered on the present moment. As a result, these children are locked into the present moment, entirely focused on their sensory self-stimulations, and this raises the question of their representations of the past or future [154].

#### 4.3.2. Melatonin and Adaptation to Change

Abnormally low daytime and nighttime melatonin secretion was associated with an absence of melatonin circadian variation in individuals with autism [59,66,77], which in turn, given the synchronizer role of melatonin, also has consequences for the circadian rhythms network This blunted circadian rhythmicity with no or little variability might be related to the difficulties in adapting to changes associated with repetitive and restricted interests typically observed in individuals with autism. Thus, we can hypothesize that children with autism who are confronted with physiological continuity due to absent circadian rhythms have difficulties to adapt to changes in either their external or their internal environment. In which case we should talk about intolerance to change, rather than a need for changelessness, or the “preservation of sameness” described by Leo Kanner [157]. For, as we saw earlier, continuity (“the endless continuum”) is as stressful as unpredictable disruptions. This underlines the importance of the circadian rhythms network synchronized by melatonin and involving an internal system of continuity/discontinuity that may participate to the developmental process of adaptation to environmental changes.

This difficulty in children with autism to adapt to change is also observed with regard to changes in the rhythm of environmental stimuli. Sometimes, this difficulty may even spark off an epileptic seizure (epilepsy is observed in nearly a third of children with autism [158]), due to the unusually rapid rhythm of a sensory stimulus, such as the pulsing light of a stroboscope. The latter can disturb the rhythmic activity of a particular brain area, leading it to fall out of sync with the rest of the brain and causing its population of neurons to fire (depolarization). It is noteworthy that individuals with autism and seizures tended to have an abnormal rhythm of melatonin correlated with EEG changes [65]. Patients with autism are particularly sensitive not only to changes in the rhythm of the external environment but also to ones occurring in their internal environment. Thus, some female adolescents with autism experience epileptic seizures towards the 14th day of their menstrual cycle, which is when luteinizing hormone (LH) levels peak. We end up with a chain of events where a change in rhythm associated with excessive environmental stimuli would strongly increase arousal and provoke physiological stress which would in turn, for some children with autism, lead to an epileptic seizure. This underscores the importance for individuals with autistic disorder of maintaining stable physiological rhythms.

## 5. Conclusions

The importance of rhythms can be applied to the calming and relaxing effect of visual and auditory stimuli featuring repeated movements with a stable rhythm, such as the ebb and flow of waves or the regular ticking of a clock… This beneficial effect opens interesting perspectives and was known about long ago in the treatment of mental illness. Thus, the former psychiatric hospital in the Syrian city of Aleppo had “treatment fountains” where the rhythm of the water flow varied according to the pathology (depression, schizophrenia, *etc.*) [159]. André Bullinger [160] is using sensory (tactile, auditory, visual, olfactory and vestibular) flows to treat children with autistic disorder, based on compensation techniques. The objective is to create a substitute flow made up of stable sequences of sensory stimuli that are regularly repeated, in order to allow children to extract invariants from a system of continuity-discontinuity. New therapeutic perspectives for autistic disorder could be developed in order to create continuity from sensory discontinuities regularly repeated with a stable rhythm, and possible moments of emotional synchrony. Thus, it appears important to introduce relational variants with emotional synchrony into a background of invariants, repeated at stable and regular intervals, making up the “holding” environment of continuity. This probably accounts for the therapeutic effectiveness of Bullinger’s technique [159], whereby a checkerboard is moved rhythmically in front of a child with autism in order to set up substitute optical flows. At the same time, the therapist looks directly at the child and attempts to share moments of emotional and relational synchrony.

To develop therapeutic new perspectives in ASD, first it is necessary to restore some basic regular physiological rhythms such as circadian rhythms. Thus, prescribing small physiologic doses of melatonin could help to restore the impaired circadian melatonin rhythm in ASD which disturbs the sleep-wake rhythm, but more generally the synchronization of internal biological clocks, resulting in the absence of homogeneous and harmonious rhythmicity with the consequences previously described on social communication, stereotyped behaviors and adaptation to environmental changes. It may well be this internal asynchrony that causes children with autism to feel permanently “out of sync” with other people. We can refer here again to René Diatkine’s patient—a high-functioning adult with autism—who explained during a filmed session that he is never in sync with others despite his efforts to adjust [155]. Randomized clinical trials in ASD are warranted to establish potential therapeutic efficacy of melatonin for social communication impairments and stereotyped behaviors or interests. In particular, studies of the dose-response relationship for melatonin in ASD are necessary in complement of dose escalation studies. We are currently conducting a therapeutic trial studying melatonin dose-effect relation in 32 male children with autistic disorder (Clinical Trials.Gov.: NCT01780883, ANSM A91245-56 [161]); the main objective of this randomized, double-blinded, placebo-controlled trial is to assess the melatonin effect on the severity of behavioral autistic impairments. Furthermore, future trials should aim at determining optimal clinical responses by the association of light treatment with melatonin. Thus, therapeutic effects might be in part mediated by enhancement of the circadian system functioning. This supports inclusion of chronotherapeutic strategies in the treatment options offered to individuals with ASD.

Finally, it might be interesting to take into account physiological rhythms, such as sleep-wake and eating rhythms, for the care provided to individuals with ASD (prescription of regular set times for eating and sleeping) and for the patient follow-up. Indeed, these physiological rhythms, in particular sleep-wake rhythms, can give important clues to look for positive trends in the follow-up.

## Figures and Tables

**Table 1:** Studies of melatonin in individuals with autism.

Study	Sample	Study group	Measured variable	Results
Ritvo *et al.* (1993) [64]	Urine	Young adults with autism (*n =* 10)	Melatonin concentration	Increased daytime values compared to typically developing controls; Similar nighttime values compared to typically developing controls
Nir *et al.* (1995) [65]	Serum	Young men with autism (*n =* 10)	Melatonin concentration	Increased daytime values compared to typically developing controls; Decreased nighttime values compared to typically developing controls
Kulman *et al.* (2000) [66]	Serum	Children with autism (*n =* 14)	Melatonin concentration (24-h circadian rhythm)	Decreased nighttime values compared to typically developing controls; No circadian variation in 10/14 (71.4%) children with autism; Inverted rhythm in 4/14 (28.6%) children with autism
Tordjman *et al.* (2005) [58]	Urine	Children and adolescents with autism (*n =* 49)	6-Sulphatoxymelatonin excretion rate (12-h collection)	Decreased nighttime values compared to typically developing controls;
Melke *et al.* (2008) [67]	Plasma	Adolescents and young adults with autism (*n =* 43)	Melatonin concentration	Decreased daytime values compared to typically developing controls
Mulder *et al.* (2010) [68]	Urine	Children and adolescents with autism (*n =* 20)	6-Sulphatoxymelatonin excretion rate (24-h collection)	Trend to lower 24-h melatonin excretion rate in hyperserotonemic compared to normoserotonemic individuals with autism
Tordjman *et al.* (2012) [59]	Urine	Postpubertal adolescents and young adults with autism (*n =* 43)	6-Sulphatoxymelatonin nexcretion rate (split 24-h collection)	Decreased daytime values compared to typically developing controls; Decreased nighttime values compared to typically developing controls No circadian variation in 10/43 (23.2%) individuals with autism

**Table 2:** Studies on potential therapeutic benefits of melatonin in autistic disorder.

Study	Journal	Population	Design	Duration of treatment	Melatonin (formulation, dose)	Time of intake	Main outcome measures	Effects on sleep	Other outcomes	Comments
Single case reports										
Horrigan and Barnhill, 1997 [81]	*J. Am. Academy Child and Adolesc. Psychiatry*	17 year old boy with Asperger’s Syndrome (AS)	_____	Not Given	3 mg	20–30 min before bedtime (BB)	Sleep	Sleep improvement. No side effects	Daytime behavior improvement	_____
Hayashi, 2000 [82]	*Psychiatry Clin. Neurosc.*	14-year-old boy with autistic disorder, severe intellectual disability and phase delay with polyphasic sleep	_____	4 months	Immediate Release (IR) 6 mg	11:00 PM	Sleep	Melatonin increased sleep duration. No side effects	None	_____
Jan *et al*., 2004 [83]	*Dev. Med.Child Neurol.*	12 year-old boy with AS and complex sleep disturbance (phase delay and parasomnias)	_____	6 months	Controlled Release (CR) 5 mg	30 min BB	Sleep	Normalization of the sleep-wake rhythm and disappearance of parasomnias. No side effects	None	_____
Retrospective studies										
Gupta and Hutchins, 2005 [84]	*Arch. Dis. Child*	9 cases of children with Autistic Disorder (AD) aged from 2–11 years. Chronic sleep problems	Not Given	1 week to 1 year	IR 2.5–5 mg	45 min BB	Parental evaluation of sleep	56% showed improvement in total sleep duration	None	No standardized collection of sleep variables
Andersen *et al.*, 2008 [85]	*J. Child. Neurol.*	107 children and adolescents aged from 2–18 years with ASD (DSM-IV): 71% AD, 5% AS, 19% PDDNOS (Pervasive Developmental Disorder Not Otherwise Specified)	Not Given	Mean Duration: 1.8 years	IR in 91% of the cases. Dose escalation protocol from 1 to 6 mg based upon age	30–60 min BB	Parental evaluation of sleep	Parents reported full (25%) or partial (60%) improvement. Beneficial effects of melatonin seem to stop after 3 to 12 months despite the use of higher doses. Side effects observed in 3 children: sleepiness, fogginess, increased enuresis	None	No standardized collection of sleep variables. The loss of response to melatonin treatment is discussed in the text
Galli-Carminatti *et al.*, 2009 [86]	*Swiss. Med. Wkly*	6 adult patients with AD (CIM-10) and intellectual disability, aged from 19–52 years	Not Given	6 months	IR. Dose escalation protocol from 3 to 9 mg if clinically required	45 min BB	Sleep (CGI-S and CGI-I)	Improvement in sleep onset latency, night and early morning awakenings. No side effects	None	No standardized collection of sleep variables. 2 to 4 associated psychotropic drugs per patient
Open label trials										
Jan *et al*., 1994 [87]	*Dev. Med. Child Neurol.*	15 children with multiple neurological disabilities and severe sleep disorders	Not Given	Not Given	2–10 mg	bedtime	Not Given	Partial improvement in sleep disorders. No side effects	Behavior and social improvement	Heterogeneous sleep disorders and neurological disabilities
Ishizaki *et al*., 1999 [88]	*No To Hattatsu*	50 children and young adults with autism (*n =* 27) or mentally retardation (*n =* 20) or severe motor/intellectual disability (*n =* 3) aged from 3–28 years with sleep disorders	Not Given	Not Given	Not Given	Not Gven	Sleep disorders and emotional/behavior disturbances	34 patients experienced improvement in response to melatonin. Side effects reported in 17 patients	Improvements in excitability when sleep also improved. No change in contrariness, stereotyped behavior and in school/workshop refusal	Various types of insomnia and diagnoses
Paavonen *et al*., 2003 [89]	*J. Child. Adolesc. Psychopharmacol.*	15 children with AS (DSM-IV) aged from 6–17 years with severe sleep problems for at least 3 months	Not Given	14 days	IR 3 mg	30 min BB	Sleep (72h-period actigraphy, sleep diaries), daytime behavior (Karolina Sleepiness Scale: KSS), Child Behavior Check List (CBCL)	Melatonin treatment was associated with significant decrease in sleep onset latency and nocturnal activity. Discontinuation of melatonin led to a significant decrease in sleep duration and more nocturnal activity. Side effects in 20% of the cases: tiredness, headaches, severe sleepiness, dizziness, diarrhoea	Significant improvement of daytime behavior (CBCL)	No principal outcome specified. KSS is not validated in children nor in ASD
Giannotti *et al.*, 2006 [90]	*J Autism Dev. Disord.*	29 children with AD (DSM-IV) aged from 2–9 years with current sleep problems	Controlled-release melatonin	6 months	Dose escalation protocol from 3 mg (1 mg of IR+2mg of CR) to 6 mg when clinically required, based upon age (max 4 mg under 4 years old and max 6mg over 6 years old)	08:00 PM	Sleep (diaries and Children Sleep Habits Questionnaire CSHQ), daytime behavior, Children Autistic Rating Scale (CARS)	Melatonin treatment was associated with improvement in sleep onset latency, night awakenings and sleep duration which vanished after melatonin discontinuation. No side effects	Parents reported less irritability, less anxiety and better mood. Significant improvement of depression, anxiety and withdrawal symptoms during melatonin treatment in children with AS. No effect was reported on the CARS	No principal outcome specified. Missing data: analyses on 25 patients
De Leersnyder *et al.*, 2011 [91]	*Pediatr. Neurolog.*	88 children with heterogeneous neurodevelopmental disorders (Smith Magenis syndrome, mental retardation, encephalopathy, Angelman syndrome, Rett syndrome, Bourneville syndrome, blindness and autism) aged 5–20 years. 7 patients with autism, mean age 12 years old	6 years of open label follow up	3 months	CR 2–4 mg (<40 kg) or 6 mg (>40 kg) based upon weight	60 min BB	Parental evaluation of sleep and mood (self-constructed questionnaire)	According to parental reports, both sleep latency and sleep duration improved within 3 months such as night awakenings, sleep quality and daytime napping. 11 children experienced adverse events (daytime nap, difficulties in swallowing tablets) that the parents attributed to melatonin treatment	12% of the parents reported improvements of mood in their children	Heterogeneous neurodevelopmental disorders. Results can’t apply to a population with autism spectrum disorders. No standardized collection of sleep and mood parameters. Mean dose for patients with autism: 5.7 mg
Malow *et al*., 2011 [92]	*J. Autism Dev. Disord.*	24 children with ASD (DSM-IV, ADOS): AD, AS and PDDNOS aged from 3–9 years. Sleep onset delay of 30 min or longer confirmed on actigraphy. Exclusion of neurodevelopmental disabilities such as fragile X, Down and Rett syndromes	Before treatment families received structured sleep education and children underwent a treatment acclimatation phase in order to be sure the melatonin will be taken	14 weeks	CR. Dose escalation protocol from 2–9 mg when clinically required	30 min BB	Sleep (actigraphy, Children Sleep Habits Questionnaire CSHQ, diaries), daytime behavior (Child Behavior Check List CBCL, Repetitive Behavior Scale-Revisited), parental stress (Parenting Stress Index Short Form), side effects (Hague Side Effects Scale)	Significant improvement in sleep latency within the first week of treatment but not for other sleep parameters such as night awakenings and sleep quality	Significant improvement in children behavior (withdrawal, affective problems, attention-deficit hyperactivity, stereotyped and compulsive behaviors). Significant improvement in parental stress	No placebo
Placebo-controlled trial										
McArthur and Budden, 1998 [93]	*Dev. Med. Child Neurol.*	9 children and adolescents with Rett syndrome aged from 4 to 17 years. Mean age :10 years old	Randomised double-blind crossover trial	2 periods of 4 weeks with a wash out period of 1 week	2.5–7.5 mg based on weight	60 min BB	Sleep (actigraphy, diaries)	Significant improvement in total sleep time. No side effects.	None	_____
Garstang and Wallis, 2006 [94]	*Child Care Health Dev.*	11 children and adolescents with ASD aged from 5–15 years with chronic sleep disorders resistant to behavioral treatment	Randomised double-blind crossover trial	2 periods of 4 weeks with a wash out period of 1 week	IR 5 mg	60 min BB	Sleep (diary)	Melatonin and placebo were associated with significantly decreased sleep latency and nocturnal awakenings, increased total sleep time. No side effects	Several parents and class teachers commented that their children were easier to manage and less rigid in their behavior while taking melatonin	ASD criteria were not consensual. Only 7 children completed the trial. Investigators found that some of the placebo capsules were empty. Missing data
Wasdell *et al.*, 2008 [95]	*J. Pineal Res.*	51 children and adolescents with neurodevelopmental disabilities (16 patients with ASD) from 2–18 years. Sleep delay phase syndrome and impaired sleep maintenance with resistant to sleep hygiene intervention	Randomised double-blind crossover trial. 3-weeks trial followed by a 3-month open-label study. Bahavioral sleep treatment before inclusion	2 periods of 10 days with a wash out period of 3–5 days	Dose escalation protocol based on unspecified conditions: from 5 mg (1 mg FR + 4 mg CR) to 15 mg	20–30 min BB	Sleep (actigraphy, diaries, CGI-S, CGI-I), familial stress (Family Stress Scale)	Significant improvement in total sleep duration and sleep latency as well as reduced stress levels in parents in the melatonin arm	Half of the patients with ASD had their dose increased during the open-label phase with no additional improvement in sleep latency or sleep duration, but caregivers reported less anxiety	Unspecified ASD criteria. 50 patients completed the trial and 47 completed the open-label phase. Selection bias due to previous melatonin treatment (25% of the cases). At the end of the trial, 29 patients received a dose of 10 or 15 mg. Higher doses were necessary in patients with bilateral cerebral lesions
Wirojanan *et al.,* 2009 [96]	*J. Clin. Sleep Med.*	12 children and adolescents with unspecified sleep problems, aged from 2–15 years: 5 patients with AD (ADOS and ADI-R), 3 patients with fragile X syndrome with AD, 3 patients with AD and fragile X syndrome, and 1 patient with fragile X premutation	Randomized double-blind crossover trial	2 periods of 2 weeks. No wash out period	IR 3 mg	30 min BB	Sleep (actigraphy, diary)	Significant, but mild improvement in total sleep time (+21min) and decrease in sleep latency (−28min)	None	Missing data: only 12 patients completed the trial (order bias). No sub-group analysis in AD patients. No side effects
Wright *et al.*, 2011 [97]	*J. Autism Dev. Disord.*	22 children and adolescents from 3–16 years with ASD (ICD-10, ADOS, ADI-R): AD (70%), AS (10%) and AA (20%). No fragile X or Rett syndrome. Current sleeplessness (confirmed on a 1 month-diary) and resistant to behavioral treatment.	Randomized double-blind crossover trial	2 periods of 3 months separated by 1 month of washout	IR. Dose escalation protocol from 2 mg to 10 mg when clinically required	30–40 min BB	Sleep (Sleep Difficulties Questionnaire, diary), daytime behavior (Developmental Behavior Checklist), Side Effect Questionnaire	Significant improvement in sleep latency (−47min) and total sleep duration (+52min) in the melatonin arm. No improvement in night awakenings. The side effect profile was not significantly different between the 2 groups	Improvement in children behavior in the melatonin arm that was significant for communication (*p* = 0.045)	Missing data. Analysis on 16 patients. No actigraphy. Mean melatonin dose: 7 mg
Cortesi *et al.*, 2012 [98]	*J. Sleep Res.*	160 children with ASD (DSM-IV, ADI-R, ADOS) aged 4–10 years with sleep onset insomnia and impaired sleep maintenance	Randomized placebo- controlled. Randomization in 4 groups: 1) melatonin alone 2) melatonin+ Cognitive Behavioral Therapy (CBT) 3) CBT alone 4) placebo	12 weeks	CR 3 mg	09:00 PM	Sleep (actigraphy, Children Sleep Habits Questionnaire, diaries)	144 patients completed the trial and 134 were analysed. Combination group showed greater significant improvements on sleep followed by the melatonin alone and the CBT alone compared to placebo group. No side effects	None	_____
Gringras *et al*., 2012 [99]	*BMJ*	146 children from 3 to 15 years with neurodevelopmental disorders (60 patients with ASD) and severe sleep disorders that did not respond to standardised sleep advice	Double-blind randomised multicentre placebo- controlled phase III trial	12 weeks	immediate release melatonin (dose escalation protocol from 0.5 mg to 12 mg) or matching placebo	45 min before bedtime	total sleep time after 12 weeks (sleep diaries and actigraphy); sleep onset latency; child behavior (aberrant behavior checklist); family functioning; adverse events	Melatonin increased total sleep time by 22.4 min (diaries) and 13.3 (actigraphy); reduced sleep onset latency by 37.5 min (diaries) and 45.3 (actigraphy). Children in the melatonin group woke up earlier than the children in the placebo group. Melatonin was most effective in children with longest sleep latency. Adverse events were similar between the 2 groups	Child behavior and family functioning outcomes showed some (but not significant) improvement and favoured use of melatonin	The results are not specified by category of developmental disorder

**Table 3:** Review, meta-analysis and discussion of therapeutic uses of melatonin in autistic disorder.

		Review/meta-analysis
Jan and O’Donnell, 1996 [100]	*J. Pineal Res.*	Review based on100 individuals with chronic sleep disorders, aged from 3 months to 21 years. Half of these 100 patients presented visual impairment or blindness. Melatonin dose ranged from 2.5 to 10 mg. Higher doses were needed in patients with impaired sleep maintenance. Partial or total improvement in sleep parameters was found in 82% of the cases. No side effects
Jan *et al*., 1999 [101]	*Dev. Med. Child Neurol.*	Systematic review of studies on melatonin in children. 24 studies found, most of them were case reports or uncontrolled studies with small samples. Mean age: 10 years old. Associated diagnosis: blindness and neurodevelopmental disabilities, 1 single case of an adolescent with AS [76]. Doses ranged from 0.5 to 20 mg. Improvement in sleep in all the studies
Phillips and Appleton, 2004 [102]	*Dev. Med. Child Neurol.*	Only three studies, reporting a total of 35 children, fulfilled the criteria for inclusion (randomized controlled clinical trials). Two of them reported a significant decrease in time to sleep onset
Braam *et al*., 2009 [103]	*Dev. Med. Child Neurol.*	Meta-analysis of placebo-controlled randomized trials of melatonin in individuals with intellectual disabilities and sleep problems. 9 studies were included. Various doses and formulations of melatonin were given. Melatonin decreased sleep latency by a mean of 34 min (*p <* 0.001), significantly decreased mean number of wakes per night (p=0.024), and increased total sleep time by 50 min (*p <* 0.001). Specified reports on adverse effects were given in four studies. Adverse effects were minor and their incidence in both melatonin and placebo phases were the same. Patient groups in studies included in this meta-analysis were very heterogeneous
Guénolé *et al*., 2011 [79]	*Sleep Med. Rev.*	Systematic review of efficacy and safety of exogenous melatonin for treating disordered sleep in individuals with autism spectrum disorders: 4 case reports, 3 retrospective studies, 2 open-label clinical trials, 3 placebo controlled trials. All studies supported the existence of a beneficial effect of melatonin on sleep in individuals with ASD with minor side effects. Limitations are: small sample, clinical heterogeneity of ASD and sleep disorders, varying methods used to measure sleep, confounding factors such as behavioral interventions and cross over design (no analysis of intention to treat). Melatonin doses ranged from 0.75 to 10 mg per day. The authors propose that future research on the efficacy of melatonin in children with ASD should include daytime functioning as a principal outcome measure. Only 6 patients on 205 presented side effects: daytime sleepiness, fogginess, dizziness, nocturnal enuresis, tiredness, headache, diarrhoea
Doyen *et al*., 2011 [78]	*Eur. Child Adolesc. Psychiatry*	Systematic review on pharmacokinetics data on melatonin and its role in sleep disorders and autism spectrum disorders. Authors reviewed 17 studies on effectiveness and side effects of melatonin in patients with AD, AS, PDD-NOS and Rett syndrome. Effectiveness on sleep disorders was found in all the studies, side effects were reported in 5 studies. Melatonin doses ranged from 0.5 to 10 mg. Melatonin seems to have anxiolytic properties. Most frequent reported side effects: infections, flu, epilepsy, intestinal disorders and agitation
Rossignol and Frye, 2011 [80]	*Dev. Med. Child. Neurol.*	Aim of the study: investigate melatonin-related findings in ASD including AD, AS, Rett syndrome and PDDNOS. 18 studies on melatonin treatment on ASD patients were identified (5 RCT), 12 of them reported improvement in sleep with melatonin in 67% to 100% of the patients. 6 studies reported improvement in daytime behavior (less behavioral rigidity, ease of management for parents and teachers, better social interaction, fewer temper tantrums, less irritability, more playfulness, better academic performance and increased alertness). Melatonin doses ranged from 0.75 to 15 mg, age of patients ranged from 2 to 18 years, treatment duration ranged from 2 weeks to 4 years. 12 studies explored side effects (headache, tiredness, dizziness, diarrhea) in which 7 studies reported no side effects. 9 studies found low levels or abnormal circadian rhythm of melatonin in ASD. A correlation between this abnormal levels and autistic behaviors was found in 4 studies. Night time urinary excretion of melatonin metabolite (6-SM) was reported to be inversely correlated with the severity of impairments in verbal communication, play and daytime sleepiness in patients with ASD. 5 studies found genetic abnormalities of melatonin receptor and enzymes involved in melatonin synthesis
Reading, 2012 [104]	*ChildCare Health Dev.*	Correlation between plasmatic levels of melatonin and autistic behaviors was found. Melatonin groups showed improvements in total sleep duration and sleep onset latency *versus* placebo groups but not on night awakenings
Letter to the editor		
Guénolé and Baleyte, 2011 [105]	*Dev. Med. Child Neurol.*	Response to the Rossignol and Frye review [73]; Authors proposed that studies should explore separately sleep disorders in patients with ASD and sleep disorders in patients with Rett syndrome
Guénolé and Baleyte, 2012 [106]	*Pediatr. neurol.*	Response to the De Leersnyder *et al*. study [86] of open label trial. The definition of « chronic sleep disorder » did not refer to international classifications. Half of the children manifested Smith-Magenis syndrome that involves specific abnormalities of melatonin secretion. Thus, results can’t apply to a population with ASD. The effects of melatonin should be studied separately in each neurodevelopmental disorder and with specific sleep diagnoses
Discussion/Commentary		
Jan and Freeman, 2004 [107]	*Dev. Med. Child Neurol.*	Discussion on melatonin use in children with ADHD, ASD, neurodevelopmental disabilities, epilepsy and blindness. Exogenous melatonin seems to regulate endogenous melatonin secretion. It seems to be more effective in sleep-wake cycle disorders with sleep onset delay disorders. Night and morning awakenings seem to be more difficult to treat, such as sleep problems associated with cerebral lesions. The more the child shows mental or motor comorbidities, the more the melatonin dose is high
Lord, 1998 [108]	*J Autism Dev. Disord*	General brief discussion of melatonin and its potential for treating sleep problems in autism
